# High-Dose siRNAs Upregulate Mouse Eri-1 at both Transcription and Posttranscription Levels

**DOI:** 10.1371/journal.pone.0026466

**Published:** 2011-10-19

**Authors:** Yingnan Bian, Wei Zhou, Yingchun Zhao, Xiaoping Li, Wei Geng, Ruixin Hao, Qing Yang, Weida Huang

**Affiliations:** 1 Department of Biochemistry, School of Life Science, Fudan University, Shanghai, China; 2 Laboratory for Synthetic Biology, Centers for Nano-Medicine, Shanghai Advanced Research Institute, Chinese Academy Sciences, Pudong, Shanghai, China; Baylor College of Medicine, United States of America

## Abstract

The *eri-1* gene encodes a 3′ exonuclease that can negatively regulate RNA interference via siRNase activity. High-dose siRNAs (hd-siRNAs) can enhance Eri-1 expression, which in return degrade siRNAs and greatly reduces RNAi efficiency. Here we report that hd-siRNAs induce mouse Eri-1 (*meri-1*) expression through the recruitment of Sp1, Ets-1, and STAT3 to the *meri-1* promoter and the formation of an Sp1-Ets-1-STAT3 complex. In addition, hd-siRNAs also abolish the 3′ untranslated region (UTR) mediated posttranscriptional repression of *meri-1*. Our findings demonstrate the molecular mechanism underlying the upregulation of *meri-1* by hd-siRNA.

## Introduction

RNA interference (RNAi) is widely accepted as a precise and effective method for specific gene knockdown in eukaryotes. It has been extensively studied as a powerful tool for experimental and therapeutic purpose. However, several unexpected effects of RNAi compromise the efficiency and spectrum of its applications. These effects include immune activation, unintended gene inhibition/activation, and potential endogenous microRNAs interference [1], [2]. Further improvements in this technology require a profound understanding of the underlying mechanisms of these obstacles.

Even RNAi delivered with high efficiency is restricted in its activity. Several negative regulatory mechanisms of RNAi have been described [3], [4], [5]. One of the best studied is the suppression of anti-viral RNA silencing by viral suppressor proteins (e.g., p19) [6]. In *C. elegans*, several negative regulators of RNAi, including *eri-1*, *eri-3*, *eri-5* and *eri-6/7*, have been identified through genetic screening [7], [8], [9]. Eri-1 is suggested to be an exonuclease that inhibits RNAi by degrading the 3′overhangs of siRNAs, which may impede the assembly of siRNA into RISCs, thus undermining the efficiency of RNAi [7]. Our previous study demonstrated that, in mice, high doses of siRNAs were less effective than low doses of siRNAs. Transcription of the mouse *eri-1* gene (*meri-1*) was upregulated by high-dose exogenous non-silencing siRNA but not by low-dose siRNA [10], and silencing of *meri-1* gene could rescue the effectiveness of RNAi [11]. These findings suggested that there was also negative regulation for the exogenous introduced siRNAs and that *meri-1* played an important role in this negative regulation. However, the exact molecular mechanism underlying the regulation is poorly understood.

Sp1 is a general transcription factor that regulates the expression of numerous genes. Both the DNA binding ability and the transactivational activity of Sp1 are influenced by posttranslational modifications such as phosphorylation, glycosylation, and acetylation [12], [13], [14], [15]. Sp1 also has multifunctional transcriptional activities through its interaction with other transcription factors, which is required for adaptive responses to differentiation [16], [17], [18], proliferation [19], and oxidative stress [12], [20], [21].

Ets-1 belongs to the Ets family that regulates gene expression in a variety of tissues and cell types [22]. This functional versatility emerges from their interactions with other structurally unrelated transcription factors [23]. Indeed, combinatorial control is a characteristic property of the Ets family members [24]. The interaction of Ets proteins with other transcription factors, including Sp1, may modify their DNA-binding properties in a promoter-specific fashion [23], [25].

STAT3 mediates a variety of biological events including inflammation, cellular transformation, survival, angiogenesis, and metastasis of cancer through collaboration with other transcription factors, including Sp1 [26], [27], [28], [29], [30]. Various types of carcinogens, radiation, viruses, growth factors, oncogenes, and inflammatory cytokines have been found to activate STAT3 [31].

In animals, when small double-stranded RNAs possess near-perfect complementarity to target sequences positioned in either coding or 3′-UTR regions of mRNAs, they can direct endoribonucleolytic cleavage of mRNA by Argonaute 2 (Ago2) through an RNAi-like mechanism induced by siRNAs [32]. However, when small double-stranded RNAs possess imperfect complementarity to the 3′-UTR of target mRNAs, they exert their repressive function by inhibiting translation initiation and/or shortening poly (A) through mechanisms that also involve AGO2 [33]. Therefore, the siRNA-mediated pathway significantly overlaps and shares the common components with the miRNA-mediated pathway [32], [33], [34], [35], which has well-recognized roles in gene regulation and may affect up to 30 percent of human genes [36]. This similarity prompted us to investigate whether hd-siRNAs also affected *meri-1* expression through interfering with the miRNA-mediated regulation in our previous study. In the miRNA pathway, after forming the miRNA-induced silencing complexes (miRISCs) on target mRNA 3′-UTR, the mRNA and miRISCs containing Argonaute proteins accumulate in discrete cytoplasmic foci known as P-bodies or GW bodies [37], [38], [39], [40], [41], [42]. This mRNA is stored or decayed in the P-bodies, resulting in posttranscriptional repression through translational repression or mRNA destabilization [35]. The miRNA pathway usually requires the recognition of multiple imperfect sites by the same or several different miRNAs and the formation of multiple miRISCs on a 3′-UTR of mRNA to get effective translational repression [43], [44], [45], [46]. Therefore, it is especially important for miRNA-mediated suppression to have sufficient miRISCs components. High-dose exogenous siRNAs may compete with endogenous miRNAs for the limited components of miRNA pathway and block the normal function of miRNA. In addition, miRNA-mediated repression can be effectively reversed or prevented under certain conditions or extracellular stimuli, which makes miRNA regulation more dynamic [35]. The repressed mRNA can also be released from P-bodies and recruited to polysomes to reactivate its translation [47].

In the present study, we analyzed the promoter region of the *meri-1* gene and its interaction with Sp1/Ets-1/STAT3. In response to the hd-siRNAs treatment, Sp1 is acetylated and binds to the *meri-1* promoter where it forms a complex with transcription factors Ets-1 and STAT3 to promote *meri-1* transcription. In addition, hd-siRNAs also abolish the 3′-UTR mediated posttranscriptional repression of *meri-1* expression. Together, these results provide key mechanisms to illustrate how hd-siRNAs induce enhanced *meri-1* expression, which is a critical determinant in reducing RNAi efficiency.

## Results

### Characterization of the *meri-1* promoter sequence that is responsive to hd-siRNA

**Figure 1:**
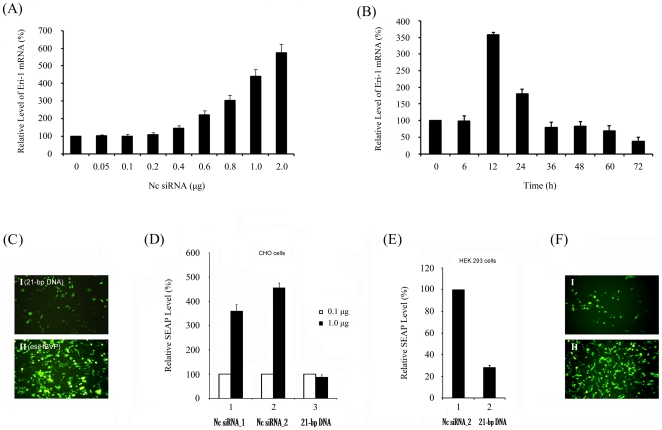
Characterization of the *meri-1* promoter Sequence. (A) Upregulation of *meri-1* transcription induced by hd-siRNAs in CHO cells. CHO cells were transfected with indicated quantity of Nc siRNA_2. The *meri-1* mRNA level was measured by qPCR, twelve hours after transfection. (B) Time course of hd-siRNA-stimulated transcription of *meri-1*. CHO cells were transfected with Nc siRNA_2. The *meri-1* mRNA level was measured by qPCR. (C) Determination of the responsive ability of the 1.8 kb *meri-1* promoter, Peri-1(−1654), to hd-siRNAs. CHO cells were co-transfected with GFP reporter construct containing the 1.8 kb *meri-1* promoter and Nc siRNA_1 (siHBVP) (II), or 21-bp DNA control (I). (D) Response of the *meri-1* promoter to enzymatically synthesized siRNA (Nc siRNA_1) and chemically synthesized siRNA (Nc siRNA_2). CHO cells were co-transfected with SEAP reporter construct containing the 1.8 kb *meri-1* promoter and Nc siRNA_1 (panel 1), Nc siRNA_2 (panel 2), or the 21-bp DNA control (panel 3). (E) Response of the *meri-1* promoter to hd-siRNA in the HEK 293 cells. (F) The necessity of liposome-mediated transfection process for the hd-siRNA-stimulated *meri-1* promoter activity. CHO cells were transfected with GFP reporter construct containing the 1.8 kb *meri-1* promoter. Four hours later, the medium was removed and the cells were washed three times by fresh medium. Then, Nc siRNA_1 was added to the medium without transfection agent (I). CHO cells were co-transfected with GFP reporter construct containing the 1.8 kb *meri-1* promoter and Nc siRNA_1 was used as a control (II).

Our previous study has showed that I.V. injection of hd-siRNAs upregulates the *meri-1* transcription in mouse liver [10]. To analyze the *in vitro meri-1* transcription in response to hd-siRNA treatment, we measured *meri-1* mRNA level by qPCR in CHO cells. As shown in Figure 1A, *meri-1* transcription was significantly upregulated in CHO cells treated with >0.4 µg/24-well plate of Nc siRNA. This *meri-1* transcription peaked at 12 h after Nc siRNA transfection and gradually decreased to its basal level after 36 h (Figure 1B). Therefore, the following experiments were carried out using 1 µg/24-well plate of Nc siRNA and data was collected 24 h after Nc siRNA transfection.

To determine whether hd-siRNAs upregulate the *meri-1* transcription through its promoter, transient transfection-based reporter gene assays were performed in CHO cells. High-dose siRNAs, either enzymatically synthesized or chemically synthesized, induced 5–7 fold of *meri-1* promoter activity when compared with the 21-bp DNA control (Figure 1C and D). Experiments in HEK 293 cells showed similar results (Figure 1E). These results suggest that the −1654/+115 region of *meri-1* promoter is significantly responsive to hd-siRNA treatment, indicating novel hd-siRNA responsive elements in this region.

To examine whether the liposome-mediated siRNA transfection procedure is necessary for *meri-1* upregulation, we first transfected the pPeri-1(−1654)-GFP reporter construct into the cells alone. Four hours later, the medium was removed and the cells were washed three times with fresh medium and treated with 1 µg/24-well plate of Nc siRNA_1. Without the transfection procedure, this direct addition of siRNA was unable to induce GFP reporter expression (Figure 1F: I), indicating that the transfection procedure was necessary for hd-siRNA to up-regulate *meri-1* transcription.

### Identification of hd-siRNA responsive elements in *meri-1* promoter

**Figure 2:**
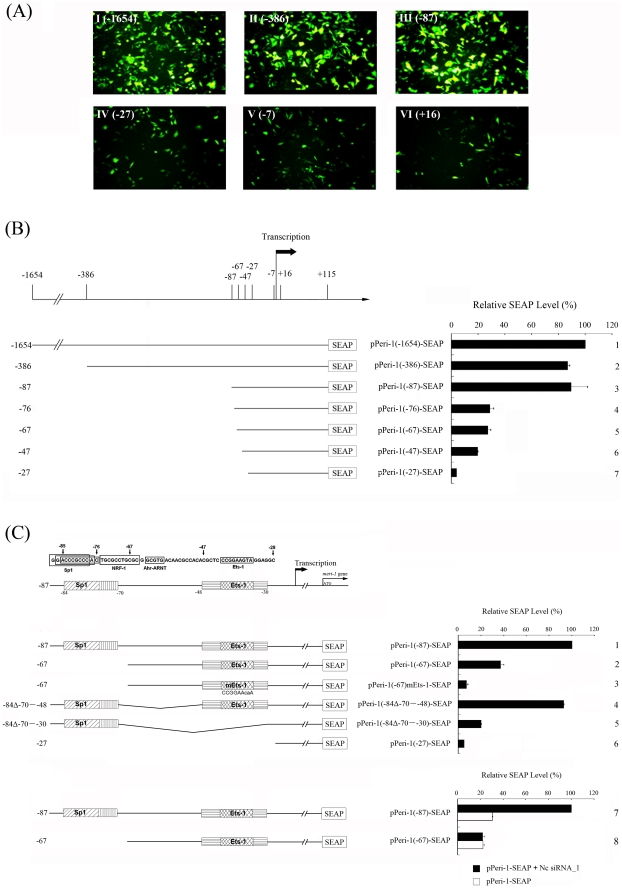
Sp1 and Ets-1 binding sites are the key *cis*-elements for the siRNA-stimulated *meri-1* promoter activity. The hd-siRNA-stimulated transcriptional activity of the truncated *meri-1* promoters was determined using the GFP reporter assay and the secreted alkaline phosphatase (SEAP) activity assay. (A) GFP assay testing the activity of the truncated *meri-1* promoters. CHO cells were co-transfected with Nc siRNA_1 and GFP reporter constructs containing the truncated *meri-1* promoters (I–VI). (B) SEAP activity assay testing the activity of the truncated *meri-1* promoters. CHO cells were co-transfected with SEAP reporter constructs containing the truncated *meri-1* promoters and Nc siRNA_1. (C) Upper panels: A schematic presentation of the putative *trans*-factor binding sites in the −87 to −28 region of the *meri-1* promoter, as predicted by MAPPER [48]. Middle panels: The SEAP activity in the culture media of the CHO cells co-transfected with Nc siRNA_1 and the SEAP reporter constructs, including the 180-bp *meri-1* promoter (Peri-1(−87)) (panel 1), the 160-bp *meri-1* promoter with deletions of Sp1 and NRF-1 site (Peri-1(−67)) (panel 2), the same fragment with a mutation in the Ets-1 binding site (Peri-1(−67)mEts-1) (panel 3), the same sequence as Peri-1(−47) but with the Sp1 site added to the 5′ end (Peri-1(−84Δ−70–−48)) (panel 4), the same sequence as Peri-1(−27) but with the Sp1 site added at the 5′ end (Peri-1(−84Δ−70–−30)) (panel 5), or the 120-bp *meri-1* promoter (Peri-1(−27)) (panel 6). Lower panel: The SEAP activity in the culture media of the CHO cells transfected with the truncated *meri-1* promoters. The CHO cells were co-transfected with SEAP reporter constructs containing the truncated *meri-1* promoters and Nc siRNA_1 or 21-bp DNA control. The data represents the mean of three independent experiments, and the error bars indicate the SD of triplicate samples.

We analyzed the *meri-1* promoter for potential hd-siRNA responsive elements as follows. Several GFP or SEAP reporter constructs driven by *meri-1* promoter fragments of various lengths were made and transfected into CHO cells. Similar reporter activity (<10% difference) upon siRNA treatment was observed for the 1.8 kb promoter (Peri-1(−1654)), the 500 bp (Peri-1(−386)), and the 200 bp fragments (Peri-1(−87)) (Figure 2A: I–III; Figure 2B: panels 1–3). However, the 140 bp fragment (Peri-1(−27)) lost most of the promoter activity (Figure 2A: IV; Figure 2B: panel 7), indicating that −87 to −27 region contains critical elements responsible for hd-siRNA stimulated *meri-1* expression.

Scanning this region with the MAPPER program (multi-genome analysis of positions and patterns of elements of regulation, http://mapper.chip.org/mapper/mapper-main) [48] revealed a potential Sp1 site (CCGCCC) at −87 to −76, a potential NRF-1 site (TGCGCCTGCGC) at −75 to −65, a potential Ahr-ARNT site (GCGTG) at −63 to −59, and a potential Ets-1 site (CCGGAAGTA) at −42 to −34 (Figure 2C). Based on this result, a series of SEAP reporter constructs carrying *meri-1* promoter with deletions in this region were constructed and the promoter activity was analyzed. As shown in Figure 2B, two deletions contribute to the decline of promoter activity: one from pPeri-1(−87) to pPeri-1(−76) containing an Sp1 binding site and another from pPeri-1(−47) to pPeri-1(−27) containing an Ets-1 binding site.

To further verify the importance of the Sp1 and Ets-1 binding sites, another series of *meri-1* promoter mutations were prepared (Figure 2 B: panel 3–4; Figure 2 C: panels 1–6). Single Sp1 binding site deletion (−87 to −76) led to significantly decreased promoter activity (Figure 2 B: panel 4). The promoter Peri-1(−84Δ−70–−48) (Figure 2 C: panel 4), which retained both Sp1 and Ets-1 binding sites, showed almost the same promoter activity as that of pPeri-1(−87). In comparison, the promoter Peri-1(−84Δ−70–−30) (Figure 2 C: panel 5), which deleted the Ets-1 binding site, exhibited dramatically decreased promoter activity. Mutation of the potential Ets-1 binding site in pPeri-1(−67)-SEAP, from CCGGAAGTA to CCGGAACAA (Figure 2 C: panel 3) result in a subsequent decreased promoter activity comparable to that of pPeri-1(−27)-SEAP construct (Figure 2 C: panel 6). In addition, deletion of the Sp1 binding site led to not only a significant decrease in the promoter activity, but also a loss of the ability to respond to hd-siRNA treatment, although limited transcriptional activity was retained in this case (Figure 2C: panels 7 and 8). Altogether, these results indicate that both Sp1 and Ets-1 binding sites are involved in the hd-siRNA stimulated *meri-1* transcription and may play different roles.

### Involvement of Sp1 and Ets-1 in the hd-siRNA-stimulated *meri-1* promoter activity

**Figure 3:**
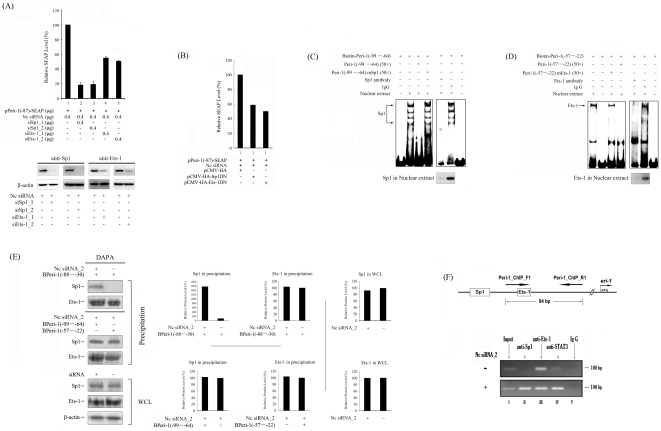
Involvement of Sp1 and Ets-1 in the siRNA-stimulated *meri-1* Promoter Activity. (A) Knockdown of Sp1 and Ets-1 genes inhibits the siRNA-stimulated *meri-1* promoter activity. A: The SEAP activity in the culture media of the CHO cells co-transfected with pPeri-1(−87)-SEAP and Nc siRNA plus siSp1_1, siSp1_2, siEts-1_1 or siEts-1_2 was measured. The knockdown effects were shown in lower panel. (B) Overexpression of the Sp1 and Ets-1 dominant-negative mutant inhibits the siRNA-stimulated *meri-1* promoter activity. The SEAP activity in the culture media of the CHO cells co-transfected with pPeri-1(−87)-SEAP and Nc siRNA plus pCMV-HA, pCMV-HA-Sp1(DN) or pCMV-HA-Ets-1(DN) was measured. The data represents the mean of three independent experiments, and the error bars indicate the SD of triplicate samples. (C)–(F) Sp1 and Ets-1 binding specifically to the *meri-1* promoter. (C) and (D) EMSAs were performed using various biotin-labeled oligonucleotides corresponding to the putative Sp1 and Ets-1 elements in the *meri-1* promoter, as described in Table 1. Nuclear extracts were prepared from CHO cells pre-treated with Nc siRNA_1. The competition assays were performed with or without a 50-fold excess of each unlabelled oligonucleotide, as indicated above each lane. Immunodepletion EMSAs were performed using 1 µl of the anti-Sp1, anti-Ets-1 antibodies and control normal mouse IgG, as indicated above each lane. Immunodepletion effects were shown under the related lanes. (E) Cell extracts of the CHO cells pre-treated with or not with Nc siRNA were prepared. The aliquots were subjected to DNA affinity precipitation assay (DAPA) with biotinylated oligonucleotides that contained the Sp1-, Ets-1- or both Sp1- and Ets-1-binding sites in the *meri-1* promoter (see Table 1). The precipitated complexes (Precipitation) were immunoblotted with anti-Sp1 or anti-Ets-1 antibodies, and the whole cell lysates (WCL) were monitored for the expression of Sp1 or Ets-1. The relative intensity of bands was quantified using the ImageGouge software (Fuji). (F) CHO cells treated with or not with hd-siRNAs were harvested for chromatin immunoprecipitation (ChIP) assays. Chromatin suspensions were immunoprecipitated with the indicated antibodies and control IgG. The resulting precipitants were subjected to PCR to amplify the region as indicated in the upper panel. The amplified products were visualized by ethidium bromide staining after gel electrophoresis. Representative data of 30 cycles are shown.

To examine the roles of Sp1 and Ets-1 in the hd-siRNA-stimulated *meri-1* promoter activity, specific siRNAs were prepared to knock down Sp1 and Ets-1. These siRNAs were used to transfect CHO cells together with hd-siRNAs and pPeri-1(−87)-SEAP reporter plasmid. SEAP assays revealed that the knockdown of Sp1 and Ets-1 significantly decreased *meri-1* promoter activity (Figure 3A). These results suggest that Sp1 and Ets-1 are involved in the regulation of hd-siRNA-stimulated *meri-1* promoter activity.

To confirm the roles of Sp1 and Ets-1 in the hd-siRNA-stimulated *meri-1* promoter activity, the dominant-negative mutant of Sp1 and Ets-1 expression vectors, pCMV-HA-Sp1 (DN) lacking the transactivation domain of Sp1 [49] and pCMV-HA-Ets-1 (DN) lacking the transcription activation domain [50], were constructed. CHO cells were co-transfected with pPeri-1(−87)-SEAP and Nc siRNA plus pCMV-HA, pCMV-HA-Sp1 (DN) or pCMV-HA-Ets-1 (DN). Expression of the Sp1 dominant-negative mutant led to an ∼40% decrease in SEAP activity (Figure 3B: panel 2). Similarly, the expression of the Ets-1 dominant-negative mutant also led to an ∼50% decrease in SEAP activity (Figure 3B: panel 3). These experiments provide further evidence supporting the key roles of Sp1 and Ets-1 in hd-siRNA-stimulated *meri-1* promoter activity.

### Sp1 and Ets-1 bind specifically to the *meri-1* promoter

To determine whether Sp1 and Ets-1 bind specifically to their binding motifs (the boxes in Figure 2C) in the *meri-1* promoter, competition EMSAs were carried out. The EMSA results (Figure 3C and D) showed that the biotin-labeled oligonucleotides coding for the Sp1 and Ets-1 binding motifs formed DNA-protein complexes with nuclear proteins. In addition, pretreatment with excess unlabelled oligonucleotides abolished the binding of nuclear proteins to the biotin-labeled oligonucleotides, whereas the addition of excess unlabeled mutant oligonucleotides did not show any obvious effects. To further confirm that Sp1 and Ets-1 bind to the putative Sp1 and Ets-1 motifs after siRNAs treatment, immunodepletion EMSAs were performed using anti-Sp1 or anti-Ets-1 antibody. The intensity of the retarded bands was decreased (immunodepletion) by anti-Sp1 antibody and anti-Ets-1 antibody (Figure 3C and D). These results indicate that Sp1 and Ets-1 can bind specifically to their respective binding motifs.

To further confirm the binding characteristics of Sp1 and Ets-1 with the *meri-1* promoter, DNA affinity precipitation assays (DAPAs) were performed using CHO cell extracts (Figure 3E). Only when the CHO cells were treated with hd-siRNA were the complex comprising Peri-1(−87), Sp1 and Ets-1 formed. Few Sp1 bound to the Peri-1(−88–−30) oligonucleotides when no siRNA induction was performed. However, the binding of Ets-1 to the Peri-1(−88–−30) oligonucleotides was siRNA-treatment-independent. In addition, cell extracts from hd-siRNAs-treated CHO cells also demonstrated the formation of a complex comprising the Peri-1(−99–−64) oligonucleotides, Sp1 and Ets-1, as well as the formation of a complex comprising the Peri-1(−57–−22) oligonucleotides, Ets-1 and Sp1. These results suggest that the hd-siRNA treatment leads to the complex formation of Sp1 with Ets-1 on promoter Peri-1 (−87).

Furthermore, ChIP assays were performed to examine these results *in vivo*. In agreement with the results of EMSAs and DAPAs, Sp1 binding to the *meri-1* promoter was significantly enhanced by hd-siRNA treatment, whereas Ets-1 binding was not changed (Figure 3F: II and III).

### Necessity of the acetylation of Sp1 in the hd-siRNA-stimulated *meri-1* promoter activity

**Figure 4:**
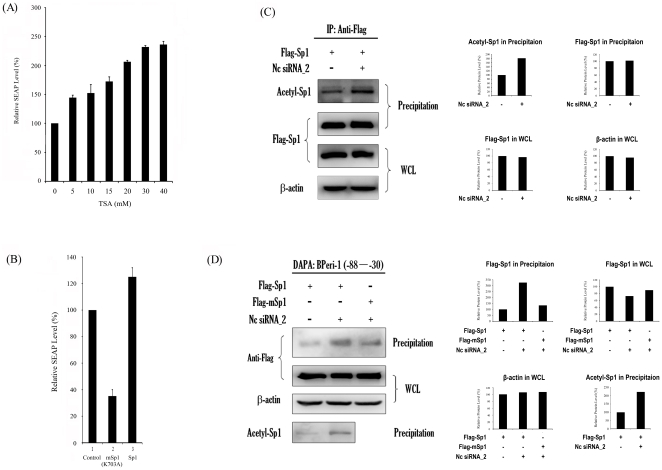
Necessity of the Sp1 acetylation in the siRNA-stimulated *meri-1* Promoter Activity. (A) The histone deacetylase (HDAC) inhibitor TSA enhances the *meri-1* promoter activity in a dosage-dependent manner. TSA was added to the culture media of the CHO cells transfected with pPeri-1(−87)-SEAP and Nc siRNA_2. (B) Mutation of the acetylation site interferes with the function of Sp1 as the activator of *meri-1* promoter. The SEAP activity in the culture media of the CHO cells co-transfected with pPeri-1(−87)-SEAP and Nc siRNA_2 plus pCMV-HA, pCMV-HA-Sp1 (K703A) or pCMV-HA-Sp1 was measured. The data represents the mean of three independent experiments, and the error bars indicate the SD of triplicate samples. (C) The acetylation of Sp1 is enhanced by hd-siRNA. Cell extracts were prepared from the CHO cells transfected with pCMV-FLAG-Sp1 or co-transfected with pCMV-FLAG-Sp1 and Nc siRNA_2. Immunoprecipitation was performed using M2-anti-FLAG antibody-agarose beads. The immunoprecipitated pellets were analyzed by immunoblotting with anti-acetyl-lysine (upper panel) and anti-FLAG (lower panel) antibodies. The relative intensity of bands was quantified using the ImageGouge software. (D) Acetylation is necessary for the recruitment of Sp1 to the *meri-1* promoter. Cell extracts of the CHO cells transfected with pCMV-FLAG-Sp1 or co-transfected with pCMV-FLAG-Sp1 and Nc siRNA_2, or with pCMV-FLAG-Sp1 (K703A) and Nc siRNA_2 were prepared. The aliquots were subjected to DAPA with biotinylated Peri-1(−88–−30) oligonucleotides that contain the hd-siRNA elements in the *meri-1* promoter (see Table 1). The precipitated complexes (Precipitation) were immunoblotted with anti-FLAG and acetyl-lysine antibodies, and the whole cell lysates (WCL) were monitored for the expression of FLAG-Sp1 or FLAG-Sp1 (K703A). The relative intensity of bands was quantified using the ImageGouge software.

Several studies have shown that Sp1 is acetylated to regulate its DNA binding affinity or transactivation [12], [51], [52], [53]. In neurons, Sp1 can be acetylated in response to oxidative stress and TSA-induced Sp1 acetylation correlated with an increase in Sp1 DNA binding [12]. To further elucidate the mechanism details of the transcriptional regulation of *meri-1*, the role of Sp1 acetylation in regulating the *meri-1* promoter activity was examined. CHO cells were treated with the prototypic histone deacetylase (HDAC) inhibitor TSA [54], [55], which is an organic hydroxamic acid that can potently inhibit the zinc hydrolase activity of HDACs by chelating zinc [56]. TSA concentration-dependently increased SEAP activity in the CHO cells transfected with hd-siRNA (Figure 4A). Since TSA might also inhibit the deacetylation of proteins other than Sp1, an Sp1 mutant expression vector, pCMV-HA-mSp1^K703A^, was constructed. It carries a mutation at lysine residue 703 of Sp1 that resides in the DNA binding domain and is the only known acetylated residue of Sp1 [52]. The overexpression of Sp1^K703A^ suppressed the transcriptional activity of *meri-1* promoter (Figure 4B). To further determine the role of Sp1 acetylation in the siRNA-stimulated *meri-1* promoter activity, the acetylation of Sp1 was assessed in hd-siRNA transfected cells. CHO cells were transfected with hd-siRNA and FLAG-Sp1 expression construct. The cell extracts were immunoprecipitated with M2-anti-FLAG antibody-agarose beads, and the immunoprecipitated pellets were blotted with anti-acetyl-lysine and anti-FLAG antibodies. As shown in Figure 4C, the acetylation of Sp1 in hd-siRNA treated cells was significantly increased compared to the control transfected cells. Next, the influence of Sp1 K703 acetylation on the recruitment of Sp1 to the *meri-1* promoter was investigated using cell extracts from the hd-siRNA-treated CHO cells. By DAPAs, as shown in Figure 4D, the mutation of the acetylated residue reduced the binding of Sp1 to the Peri-1(−88–−30) oligonucleotides. This data suggests that the acetylation of Sp1 K703 is critical for the recruitment of Sp1 to the promoter of *meri-1* induced by hd-siRNA and that the acetylation of Sp1 is required for its ability to regulate the *meri-1* promoter activity.

### The function of STAT3 as a bridge between Sp1 and Ets-1 in the hd-siRNA-stimulated *meri-1* promoter activity

**Figure 5:**
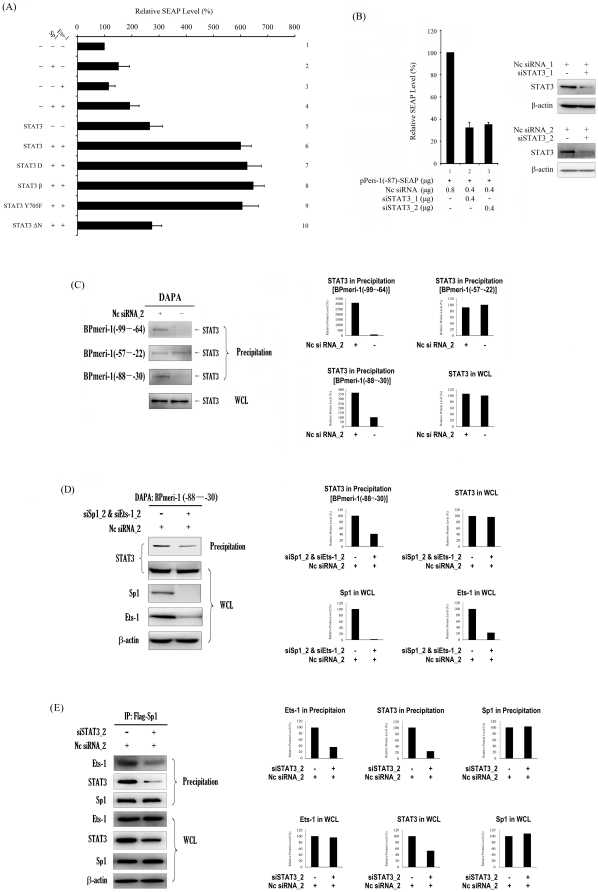
STAT3 bridges Sp1 and Ets-1 in the siRNA-stimulated *meri-1* Promoter Activity. (A) The siRNA-stimulated *meri-1* promoter activity is further enhanced by overexpression of STAT3. By using various STAT3 mutants, the involvement of STAT3 was examined. The SEAP activity in the culture media of the CHO cells co-transfected with pPeri-1(−87)-SEAP and Nc siRNA_2 plus the indicating plasmids expressing Sp1, Ets-1, STAT3 and various STAT3 mutants. (B) Knockdown of STAT3 inhibits the siRNA-stimulated *meri-1* promoter activity. The SEAP activity in the culture media of the CHO cells co-transfected with pPeri-1(−87)-SEAP and Nc siRNA plus siSTAT3_1 or siSTAT3_2 was measured. (C) STAT3 is recruited to the *meri-1* promoter by hd-siRNA induction. Cell extracts of the CHO cells transfected with or without hd-siRNA were prepared. The aliquots were subjected to DAPA with biotinylated oligonucleotides that contained the Sp1-, Ets-1- or both Sp1- and Ets-1-binding sites in the *meri-1* promoter (see Table 1). The precipitated complexes (Precipitation) were immunoblotted with anti-STAT3 antibody, and the whole cell lysates (WCL) were monitored for the expression of STAT3. The relative intensity of bands was quantified using the ImageGouge software. (D) STAT3 functions in the *meri-1* promoter activity without DNA binding. Cell extracts of the CHO cells transfected with Nc siRNA_2, or with Nc siRNA_2 plus siSp1_2 and siEts-1_2 were prepared. The aliquots were subjected to DAPA with biotinylated Peri-1(−88–−30) oligonucleotides that contain the hd-siRNA elements in *meri-1* promoter (see Table 1). (E) STAT3 functions as a bridge of Sp1 and Ets-1 in the hd-siRNA-stimulated *meri-1* promoter activity. Cell extracts were prepared from the CHO cells co-transfected with pCMV-FLAG-Sp1 and Nc siRNA_2 plus siSTAT3_2 or not. Immunoprecipitation was performed using M2-anti-FLAG antibody-agarose beads. The immunoprecipitated pellets were analyzed by immunoblotting with anti-Ets-1 antibody. The relative intensity of bands was quantified using the ImageGouge software.

Because overexpression of Sp1, Ets-1 or Sp1 and Ets-1 could only increase *meri-1* transcription very slightly (Figure 5A: panels 2–4) , the later studies, which have shown that siRNAs can activate innate immunity in mammalian cells [57], [58], [59], [60], [61], prompted us to examine the involvement of inflammation-related transcription factors in the regulation of the hd-siRNA-stimulated *meri-1*expression. Through screening by RNAi, we found that the STAT3 knockdown significantly decreased the *meri-1* promoter activity (Figure 5B: panel 2 and 3), while the overexpression of STAT3 significantly increased the *meri-1* promoter activity (Figure 5A: panel 5). Co-expression of Sp1, Ets-1 and STAT3 increased the *meri-1* promoter activity to a higher level than Sp1 and/or Ets-1 alone (Figure 5A: panel 6), suggesting that STAT3 is essential for the siRNA-stimulated *meri-1* promoter activity.

To determine if STAT3 is directly involved in the regulation of *meri-1* promoter activity, DAPAs were performed using CHO cell extracts (Figure 5C). Only when CHO cells were treated with hd-siRNAs (siRNA_1 or siRNA_2) was the existence of STAT3 observed in the complex formed on the Peri-1(−99–−64) oligonucleotides, which contain the Sp1 binding site only. If no siRNA induction was performed, no STAT3 in the complex formed on the Peri-1(−99–−64) oligonucleotides. Although the recruitment of STAT3 to the Peri-1(−57–−22) oligonucleotides (Ets-1 binding site only) is siRNA-treatment-independent, the recruitment of STAT3 to the Peri-1(−88–−30) oligonucleotides, which includes both the Sp1 binding site and the Ets-1 binding site, was significantly increased by siRNA treatment. In the ChIP assays, the recruitment of STAT3 to the *meri-1* promoter was enhanced by hd-siRNA treatment (Figure 3F: IV).

In order to examine the role of STAT3 in regulating the *meri-1* promoter, Sp1 and Ets-1 expression vectors were co-transfected with various STAT3 mutant expression vectors, including STAT3β, STAT3 (Y705F), STAT3D and STAT3ΔN (162–770). STAT3β retains the tyrosine residue at position 705 critical for dimerization but lacks a serine residue at position 727 which is a phosphorylation site [62]. STAT3 (Y705F) carries phenylalanine substitution at Tyr705 [63]. STAT3D contains mutations at positions important for DNA binding [63]. STAT3ΔN (162–770) lacks the N-terminal residues from position 1 to 161, which were demonstrated to be critical for the nuclear translocation of STAT3 [29], [64]. Only the N-terminal deletion mutant affected the activity of STAT3 in the hd-siRNA-stimulated *meri-1* promoter activity (Figure 5A: panels 7–10). These results suggest that STAT3 might function as a part of the complex regulating the *meri-1* promoter without binding to the DNA. To further confirm this presumption, DAPAs were performed. As shown in Figure 5D, when Sp1 and Ets-1 were knocked down by RNAi, the presence of STAT3 in the complex formed on the Peri-1(−88–−30) oligonucleotides was reduced. Furthermore, when STAT3 was knocked down by RNAi, fewer Ets-1 proteins were co-immunoprecipitated with Sp1 (Figure 5F), indicating that STAT3 is a determinant of hd-siRNA-stimulated Sp1 and Ets-1 complex formation. These results suggest that STAT3 might function as an adapter protein bridging Sp1 with Ets-1 and enhancing the formation of the pre-initiation complex to activate transcription of *meri-1* gene.

### Involvement of the 3′-UTR in the upregulation of *meri-1* expression stimulated by hd-siRNA

**Figure 6:**
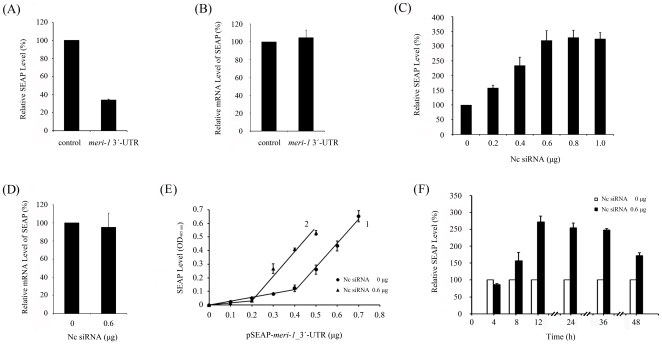
3′-UTR involved in the upregulation of *meri-1* expression stimulated by hd-siRNA. (A) 3′-UTR of *meri-1* functions in the regulation of *meri-1* expression. The SEAP activity in the culture media of the CHO cells transfected with SEAP constitutive expression construct, pSEAP2-control, or with SEAP reporter construct containing the 3.9 kb *meri-1* 3′-UTR was measured. (B) Quantity of SEAP mRNA unaffected by *meri-1* 3′-UTR. The SEAP mRNA level was measured by qPCR. (C) Nc siRNAs reduced the *meri-1* 3′-UTR-mediated repression in a dosage-dependent manner. The SEAP activity in the culture media of the CHO cells co-transfected with pSEAP-*meri-1*_3′-UTR and an increasing dose of Nc siRNA_2 was measured. (D) Quantity of SEAP-*meri-1*_3′-UTR mRNA unaffected by hd-siRNA treatment. The SEAP-*meri-1*_3′-UTR mRNA level was measured by qPCR. (E) Abolishment of the *meri-1* 3′-UTR-mediated repression by hd-siRNA treatment. The SEAP activity in the culture media of the CHO cells that were transfected with an increasing dose of pSEAP-*meri-1*_3′-UTR plus Nc siRNA_2 (line2) or not plus Nc siRNA_2 (line1) was measured. (F) Time course of the abolishment of the *meri-1* 3′-UTR-mediated repression by hd-siRNA treatment. The SEAP activity in the culture media of the CHO cells transfected with pSEAP-*meri-1*_3′-UTR plus Nc siRNA_2 or not plus Nc siRNA_2 was measured at the indicated hours after transfection.

To examine whether the 3′-UTR participates in the regulation of *meri-1* expression, equal molar amounts of the reporter plasmid, pSEAP-*meri-1*_3′-UTR (0.3 µg/24-well plate), and control plasmid, pSEAP2-control (0.17 µg/24-well plate), were transfected into CHO cells. As shown in Figure 6A, the expression of SEAP was significantly repressed by the 3′-UTR of *meri-1*. This indicates that the 3′-UTR of *meri-1* mediates the gene repression. There were no differences in the quantity of SEAP mRNA from constructs with or without the *meri-1* 3′-UTR as assessed by qPCR (Figure 6 B). This indicates that the regulation is posttranscriptional and works in an expression repression manner rather than an mRNA destabilization manner. To determine whether hd-siRNAs affected this repression, CHO cells were cotransfected with 0.3 µg of pSEAP-*meri-1*_3′-UTR and increasing doses of Nc siRNA_2. Repression was reduced by Nc siRNA in a dosage-dependent manner until a limit at approximately 0.6 µg/24-well plate (Figure 6C). The mRNA level of SEAP-*meri-1*_3′-UTR was unchanged by hd-siRNA treatment, as assessed by qPCR assay (Figure 6D), suggesting that the effect of hd-siRNA occurs at the posttranscription level. To elucidate more details of this process, we transfected a linear increasing dose of pSEAP-*meri-1*_3′-UTR with or without 0.6 µg Nc siRNA. As shown in Figure 6E, the increase of SEAP expression was not proportional to the dose of reporter plasmid (Figure 6E: line 1). A threshold of the repression effect, at about 0.4 µg/24-well plate of pSEAP-*meri-1*_3′-UTR, appeared, below which the expression of SEAP was repressed. However, with hd-siRNA treatment (Figure 6E: line 2), the inflection point appeared earlier than compared with no siRNA treatment (Figure 6E: line 1). This result confirmed that hd-siRNA could abolish the repression of *meri-1* expression exerted from its 3′-UTR. Time course analysis showed that the increase of reporter gene expression was in a time-dependent manner until it peaked at 12 h after Nc siRNA transfection (Figure 6F). Because the first four hours likely was the process of transfection, the results indicate that hd-siRNA exerted its effect rapidly. These results suggested that hd-siRNA could also abolish the posttranscription repression exerted from 3′-UTR, which further enhanced the expression of *meri-1*.

## Discussion

This study elucidated that the proximal promoter of *meri-1* contains potential hd-siRNA responsive elements and that acetylated Sp1, Ets-1 and STAT3 participate in the transcription of *meri-1*. Based on our results, we propose that, in response to treatment with hd-siRNA, Sp1, STAT 3 and Ets-1 form a complex on the *meri-1* promoter to elevate the level of *meri-1* transcription. Notably, these *trans*-acting factors play different roles in the regulation of *meri-1* transcription. Sp1 and STAT3 are responsible for the enhancement of *meri-1* transcription in response to hd-siRNA treatment, whereas Ets-1 is likely to participate in both the induced expression and constitutive expression of *meri-1*. The constitutive expression of Eri-1 has been reported in the nematode worm and in humans [7], [65]. In the nematode worm, Eri-1 is generally expressed at low levels [7]. Consistent with these reports, we found that the *meri-1* gene was still expressed at a low level without the hd-siRNA treatment. Our results suggest that Ets-1 may be involved in this constitutive expression.

It is now widely recognized that acetylation is a crucial posttranslational modification for regulation of Sp1 activity. Many findings indicated that acetylation may increase Sp1-mediated transcription [66]. The only acetylation site of Sp1 is located in the DNA binding domain [52], which suggests that acetylation of Sp1 may affect its DNA binding and/or gene transactivation functions. Previous studies reported that Sp1 was acetylated by the acetyltransferase p300, and that the interaction between the histone acetyltransferase (HAT) region of p300 and the DNA-binding domain (DBD) of Sp1 stimulated the DNA-binding activity of Sp1 [52], [67]. In our study, we found that Sp1 acetylation coincided with the increased affinity of Sp1 for DNA binding. However, the functional relevance of this post-translational modification in the DNA binding activity of Sp1 is disputed. Contradictory results were reported from other groups adopting different systems for study. Schnur *et al.* found that inhibition of HDACs increases 5-LO gene expression via enhanced Sp1/Sp3 binding to the 5-LO promoter [68]. A similar result was found in HDAC inhibitor-induced CD20 gene transactivation, in that promoter acetylation coincided with the enhanced binding of Sp1 [69]. In another study, TSA induced transforming growth factor β type II receptor (TβRII) promoter activity and acetylation of Sp1 by recruitment of HATs to the Sp1·NF-Y complex, whereas, Sp1 binding to the TβRII promoter was not changed [53]. Conversely, Waby *et al.* showed that acetylated Sp1 loses p21- and bak-promoter-binding function *in vitro*, allowing access to the promoter region by Sp3 to drive transcription in a colon cell line [15]. In the present study, the interaction of Sp1 with the *meri-1* promoter was shown to be enhanced by hd-siRNAs treatment via several independent lines of evidence. The EMSAs, DAPAs and ChIP assays demonstrated that Sp1 interacted with *meri-1* promoter after cells were treated with hd-siRNAs. We also first examined the DNA binding activity of mutant Sp1 (K703A) by DAPA. Results showed that the recruitment of Sp1 to the *meri-1* promoter was attenuated when the lysine-703 was substituted by alanine. Meanwhile, the overexpression of mutant Sp1 (K703A) significantly repressed reporter gene expression in CHO cells. This evidence indicates that the acetylation is critical for *meri-1* expression induced by hd-siRNAs. Based on the results presented in the current studies, potential roles of acetylation in regulating Sp1 DNA binding activity may be proposed. One possibility is that the acetylation is a prerequisite for Sp1 binding to the *meri-1* promoter, which may modify its biological activity including altering protein-protein interactions, DNA recognition, or/and protein stability. Acetylation might induce a conformational change in the DBD of Sp1 resulting in an increase in its affinity for DNA binding or selecting the partner proteins interacting with Sp1 which may further enhance the transcriptional competence of Sp1 and the stability of transcriptional complex. The second possibility is that the acetylation is just a concomitant consequence of Sp1 recruiting HAT to the *meri-1* promoter to activate the gene expression. It has been reported that the HAT region of p300 interacts with the DBD of Sp1 to stimulate Sp1 DNA binding activity by physical interaction rather than acetylation, despite Sp1 being acetylated by the p300 acetyltransferase region. Once Sp1 is acetylated and binds to DNA, the affinity for p300 and Sp1 was reduced [52], [67]. In our study, we found that the mutation of acetylation site compromised the binding of Sp1 to DNA. For mutant Sp1 (K703A), which cannot be acetylated, the DBD of Sp1 is continuously occupied by p300 HAT domain, which may hinder Sp1 from binding to DNA. This conclusion was supported by Hung *et al.* reported data that p300 interacted with mutant Sp1 (K703A) more than with wild-type Sp1 [52].

In this study, we found that the transactivational activity of STAT3 was independent of the phosphorylation and DNA binding activity in the hd-siRNA-stimulated *meri-1* promoter activity. This finding is distinct from the classical concept that STAT3 should bind to its own consensus DNA element. Similar to this finding, STAT3 also participated in the regulation of DINE (Damageinduced neuronal endopeptidase) without phosphorylation and DNA binding [29]. Our study further confirmed the role of STAT3 function as a necessary component of the pre-initiation complex to bridge other transcriptional components in response to stimuli. It will be of great interest to see if this molecular mechanism may be a general phenomenon in many other STAT3-regulated gene transcription systems. More evidence suggests that coactivation is an important and general mechanism in transcriptional regulation. The interaction between different *trans*-acting factors could give them functional versatility and ensure the cells' proper response to redundant stimuli by integrating multiple signals. *Trans*-acting factors activated by different pathways construct a gene-specific architecture of the transcription complex to mediate the selective control of transcriptional activity. The present study revealed that there was a function synergy of different *trans*-acting factors in the complex. In addition to STAT3 functioning as a bridge, Sp1 might function as a recruiter or scaffold for other transcription factors in the complex. Consistently, some studies have revealed that STAT3 or c-Jun can activate the promoters without their binding sites, through the interaction with Sp1 [27], [28], [29], [70], [71]. Meanwhile, Sp1 could also recruit HATs and HDACs to the promoter region to modulate transcription by directly acetylating/deacetylating the transcription factors and associated cofactors [52], [68]. Our result showed that, in the case of hd-siRNA-stimulated *meri-1* promoter activity, the acetylation of Sp1 was enhanced by Nc siRNA treatment, which suggested that the overall acetylation status of the *meri-1* promoter, which is maintained through a balance of HAT and HDAC activities, is pro-acetylation. It has been reported that the NH_2_-terminal acetylation of STAT3 is necessary for IL-6-dependent gene transcription and the decrease of HDAC1 reduced the nuclear export of STAT3, leading to nuclear accumulation of acetyl STAT3 and enhanced target gene transcription [72]. In our study, we also observed that the binding of HDAC1 to the *meri-1* promoter was impeded by hd-siRNA treatment (data not shown) and that NH_2_-terminal deletion of STAT3 suppressed the *meri-1* promoter activity. Together, we conclude that Sp1 regulates *meri-1* promoter activity not only by its transcriptional activity, but also regulating the steady state level of HATs and HDACs to control the reversible acetylation of the non-histone protein STAT3. Consequently, the subcellular distribution and nucleocytoplasmic shuttling of STAT3 dictated the duration and degree of gene activation in response to hd-siRNA.

**Figure 7:**
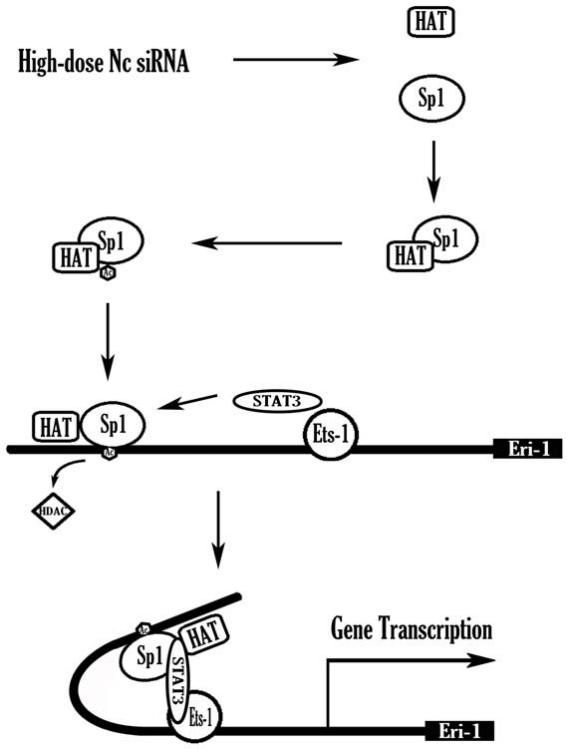
Schematic summary of the hd-siRNA-stimulated *meri-1* promoter activity. High-dose siRNAs treatment induces the interaction of Sp1 with histone acetyltransferase (HAT) leading to the acetylation of Sp1 and the DNA binding of Sp1 to the *meri-1* promoter. In addition, histone deacetylases (HDAC) are released from the promoter. STAT3 will be recruited to the *meri-1* promoter and bridge Sp1 with the co-activator Ets-1 to form the transcriptional complex and enhance the transcription of *meri-1*.

In conclusion, as shown in Figure 7, we propose that, in response to hd-siRNA treatment, Sp1 interacts with histone acetyltransferases (HAT) leading to the acetylation of Sp1 and the DNA binding of Sp1 to the *meri-*1 promoter. This occurs in conjunction with release of HDACs from the promoter. Then, STAT3 is recruited to the *meri-1* promoter and bridges Sp1 with the co-activator Ets-1 to form the transcriptional complex and elevate the level of gene transcription.

We have begun to realize the important role of the 3′-UTR of mRNA, with miRNA activity playing a major role in gene regulation [35]. In human, up to 30 percent of genes may be regulated by this mechanism [36]. In our study, we found that the *meri-1* gene is regulated by a 3′-UTR-mediated posttranscriptional repression. The expression of the reporter gene was repressed when the 3′-UTR of *meri-1* was downstream of the reporter gene. Moreover, the increase of reporter gene expression was not proportional with the dose of reporter gene transfected. When the amount of reporter vector was lower than 0.4 µg per 24-well plate, the expression of reporter gene was almost completely repressed. A significantly dosage-responsive increase emerged when the amount of reporter vector transfected was over 0.4 µg per 24-well plate. This threshold of the repression effect indicates that the amount of the components exerting repression is limited. Considering that miRNA and siRNA pathways share common components [35], we reasoned that high-dose exogenous siRNAs may compete with endogenous miRNAs for the limited components of miRNA pathway and block the normal function of miRNA. Consistent with this hypothesis is our finding that the repression of the reporter gene was abolished by Nc siRNAs in a dosage-dependent manner. In addition, in our experiments, similar results were observed when different Nc siRNAs were used (data not shown), suggesting that the *meri-1* 3′-UTR-mediated expression repression could be abolished by hd-siRNA in a sequence-independent manner. However, although the abolishment of the repression occurred rapidly after siRNA treatment and the effect of hd-siRNAs is sequence-independent, it couldn't exclude the possibility that hd-siRNAs decrease the expression of miRNAs which regulate the expression of *meri-1*. More details of the mechanism under hd-siRNA abolishing the 3′-UTR- mediated repression of *meri-1* wait to be elucidated in further studies.

RNAi is a powerful research tool, and the clinical potential of RNAi as therapeutics has been demonstrated. Some studies have demonstrated clearly that the efficacy of RNAi is based on multiple factors. Some key mechanistic aspects need to be investigated before RNAi becomes a real therapeutic modality. The amount of siRNA used is a crucial factor, as the use of non-optimal siRNA minimizes the knockdown effect [10], [11], [73], [74]. Persengiev *et al.* [57] have shown that over 1000 genes involved in diverse cellular functions were affected by conventional siRNAs in a concentration-dependent manner. Our present and previous studies indicated that, in addition to the stimulation of the mammalian innate immune response, hd-siRNAs also induced a feedback mechanism of RNAi leading to the negative regulation of RNAi efficiency [10], [11], [74]. These results caution against the application of hd-siRNA. In cases where high-dose application is necessary, especially when systemically administering combined siRNAs to multiple targets, we should inhibit the efficiency of Eri-1 to obtain an expected efficiency. The present study has provided information elemental and necessary for RNAi application.

## Materials and Methods

### Plasmids

The *meri-1* promoter fragments of various lengths were generated by PCR amplification from the genomic DNA of C57BL/6 mice, using primer P+115 (5′-cGAATTCGCCGCCTCTCGCGGGCCGCGAGAC-3′) as the antisense primer and the following primers as the sense primers, including P−1654 (5′-cgAGATCTCGGGATCCATTAATGGGCTGGTG-3′), P−386 (5′-cgAGATCTATTAATGGAATCTAGACCCTAGGAACGC-3′), P−87 (5′-cgAGATCTATTAATGGACCCGCCCACTGCGCCTGC-3′), P−76 (5′-cgAGATCTCTGCGCCTGCGCGGCGTGACAAC-3′), P−67 (5′-cgAGATCTCGCGGCGTGACAACGCCACAC-3′), P−47 (5′-cgAGATCTCGCTCCCGGAAGTAGGAGGC-3′), P−27 (5′-cgAGATCTATTAATGTCCGACGGAGTTTGTGTGG-3′), P−7 (5′-cgAGATCTATTAATCGGCCGCCGCGGGAACGTG-3′), P+16 (5′-cgAGATCTATTAATCGCGGTAGTTTTTCAAGGGAG-3′), P−84Δ−70–−48 (5′-cgAGATCTCCCGCCCACTGCGCCGCTCCCGGAAGTAGGAGGCG-3′), and P−84Δ−70–−30 (5′-cgAGATCTCCCGCCCACTGCGCGTCCGACGGAGTTTGTGTGGC-3′). Each primer was named according to the first nucleotide relative to the transcription start site (+1) of *meri-1* gene. These fragments were cloned into the pEGFP-N2 vector (Clontech, USA) between *Ase* I and *Eco*R I sites to replace the CMV promoter upstream of the GFP reporter gene. Similarly, these fragments were also cloned into the pSEAP2-control vector (Clontech, USA) between *Bgl* II and *Eco*R I sites to replace the SV40 early promoter upstream of the SEAP reporter gene. A consensus binding site mutant corresponding to Ets-1 (nt −42 to −34; 5′- CCGGAAGTA -3′ to 5′- CCGGAACAA -3′) was constructed using the QuikChange site-directed mutagenesis kit (Stratagene, USA). The 3866 bp *meri-1* 3′-UTR was cloned from the genomic DNA of C57BL/6 mice and inserted into the pSEAP2-control vector downstream of SEAP reporter gene to produce the pSEAP-*meri-1*_3′-UTR vector.

The cDNA fragments coding for Sp1, Ets-1, STAT3, dominant-negative mutant of Sp1 (Sp1DN) (amino acids 592–758), dominant-negative mutant of Ets-1 (Ets-1DN) (amino acids 306–441), STAT3ΔN (amino acids 162–770), and STAT3β were PCR amplified from their cDNAs and subcloned into the pCMV-HA vector (Clontech, USA). STAT3D and STAT3 (Y705F) mutants were generated by using the QuikChange II site-directed mutagenesis kit. The Sp1 expression vector pCMV-HA-mSp1 (K703A) carrying the Sp1 mutant (K703A) was similarly generated. All these constructs were verified by sequencing. Sp1, mSp1 (K703A) and STAT3 were also subcloned into pCMV-Tag2 (Stratagene, USA) to generate the N-terminal FLAG tagged protein.

### Preparation of siRNAs

Both enzymatically synthesized siRNAs and chemically synthesized siRNAs were used in this study. The enzymatically synthesized siRNAs for Sp1, Ets-1, and STAT3 were prepared as previously described [75], [76], and named as siSp1_1, siEts-1_1, and siSTAT3_1. They target exons 4 and 5 of mouse Sp1 gene (from 1801 to 2032 in mRNA sequence, NM_013672), exons 5 and 6 of mouse Ets-1 gene (from 876 to 1210 in mRNA sequence, NM_011808), and exon 21 of mouse STAT3 (from 2168 to 2381 in mRNA sequence, NM_213660). The chemically synthesized siRNAs include the siSp1_2 duplex 5′-GGAACAGAGUGGCAACAGUdTdT-3′ and 5′-ACUGUUGCCACUCUGUUCCdTdT-3′ [77], the siEts-1_2 duplex 5′-GGACAAGCCUGUCAUUCCUdTdT-3′ and 5′-AGGAAUGACAGGCUUGUCCdTdT-3′ [77], and the siSTAT3_2 duplex 5′-CTTCAGACCCGCCAACAAAdTdT-3′ and 5′- TTTGTTGGCGGGTCTGAAGdTdT -3′ [78].

The enzymatically synthesized nonsilencing control siRNA (esiHBVP) was derived from the Hepatitis B Virus P protein and prepared as previously described [74] and named as Nc siRNA_1. A commonly used chemically synthesized nonsilencing control siRNA (sense: 5′-UUCUCCGAACGUGUCACGUdTdT-3′, anti-sense: 5′-ACGUGACACGUUCGGAGAAdTdT-3′) was also used as Nc siRNA_2 [79], [80], [81], [82], [83], [84], [85], [86], [87], [88]. A 21-bp DNA control, the structure of which simulates siRNA duplex (sense: 5′-CGTACGCGGAATACTTCGATT-3′, anti-sense: 5′- TCGAAGTATTCCGCGTACGTT-3′), was used as a negative control in experiments where indicated.

### Cell culture and Transfection

CHO-K1 (Chinese Hamster Ovary, ATCC strain CCL 61) cells and HEK293 (Human embryonic kidney, ATCC strain CRL1573) cells were grown in Dulbecco's modified Eagle's medium supplemented with 10% fetal calf serum (Gibco, USA), 100 µg/ml streptomycin, and 100 IU/ml penicillin, at 37°C and 5% CO_2_. The cells were transfected at 70% confluence using Lipofectamine 2000 (Invitrogen, USA).

### Reverse transcription (RT)

#### Polymerase chain reaction (PCR) and quantitative real-time PCR (qPCR) Assay

Total RNA was isolated from CHO cells transfected with or without Nc siRNAs using a Qiagen RNA isolation kit. First-strand cDNA was synthesized from 1 µg of total RNA in a final volume 10 µl using ReverTra Ace® qRCP RT kit (Toyobo, Japan). The qPCR was performed using an ABI Prism 7900HT (Applied Biosystems, USA) and data were analyzed with SDS 2.2 software (Applied Biosystems, USA). All primers were designed using Primer3 (http://fokker.wi.mit.edu/primer3/) [89]. PCR amplifications (in triplicates) were carried out in a 10 µl reaction volume using THUNDERBIRD ™ SYBR® Green/ROX Realtime PCR Master Mix. The reaction conditions were as follows: 95°C for 5 min; 40 cycles at 95°C for 15 s and 60°C for 1 min; 1 cycle at 95°C for 1 min, 55°C for 30 s and 95°C for 30 s for the dissociation curve. The expression of *meri-1* was normalized to β-actin. Threshold cycle numbers (Ct) were determined with SDS 2.2 software and transformed using by the ΔCt comparative method. After PCR amplification, a melting curve of each amplicon was determined to verify its accuracy.

### Reporter Assay

GFP expression was detected as following. CHO cells were transfected with the pPeri-1-GFP series of reporter vectors together with either the Nc siRNAs or the 21-bp DNA control. Twenty-four hours later, GFP expression was measured under a LEICA DM RA2 microscope (objective 20×) at 488 nm.

Alkaline phosphatase expression was detected as following. CHO cells were transfected with the pPeri-1-SEAP series of reporter constructs together with either the Nc siRNAs or the 21-bp DNA control. Twenty-four hours later, the SEAP (secreted alkaline phosphatase) activity of the culture media was measured by performing a colorimetric assay as recommended [90]. Briefly, 50 µl of 4-fold diluted, heat-treated (65°C, 30 min) culture medium was added to 150 µl of the SEAP assay solution (20 mM para-Nitrophenylphosphate (pNPP), 1 mM MgCl_2_, 10 mM L-homoarginine, and 1 M diethanolamine, at pH 9.8). The reaction was incubated at 37°C for 15 min and the absorbance was measured at 405 nm using a microplate reader. Parallel experiments using a SEAP constitutive expression construct, pSEAP2-control, were performed to monitor and normalize transfection efficiency.

### Electrophoretic Mobility Shift Assays (EMSAs)

CHO cells were transfected with Nc siRNAs. Twelve hours after transfection, the nuclear extracts of the treated cells were prepared using the Nuclear and Cytoplasmic Protein Extraction Kit (Beyotime).

Twenty femtomoles of 5′ biotinylated double-stranded oligonucleotides (Invitrogen) were added to 2 µg of the nuclear extracts in a final volume of 10 µl that contained 0.5 µg of poly(dI-dC), 10 mM Tris-HCl (pH 7.5), 50 mM KCl, 0.5 mM DTT, 4% glycerol, 5 mM MgCl_2_ and 1% NP-40. The reactions were then incubated for 15 min at room temperature. The oligonucleotides used for EMSAs are listed in Table 1. The samples were electrophoresed at 8 V/cm through 6% non-denaturing polyacrylamide gels pre-equilibrated in 0.5×TBE (45 mM Tris, 45 mM Boric Acid and 1 mM EDTA, pH 8.3). The binding reactions were transferred to Nylon membranes and the transferred DNA was cross-linked to the membrane using the UV-light cross-linker. Finally, biotinylated double-stranded probes were detected by chemiluminescence.

**Table 1:** The oligonucleotides that were used for EMSA and Oligonucleotide Precipitation Assay.

BPeri-1 ((−99)–(−64))	5′- BIOTIN-AGAAAAAGGAGGGGACCCGCCCACTGCGCCTGCGCG-3′
Peri-1 ((−99)–(−64))	5′-AGAAAAAGGAGGGGACCCGCCCACTGCGCCTGCGCG-3′
Peri-1 mSp1F	5′-AGAAAAAGGAGAAAAAAAAAAAACTGCGCCTGCGCG-3′
BPeri-1 ((−57)–(−22))	5′-BIOTIN-CAACGCCACACGCTCCCGGAAGTAGGAGGCGTCCGA-3′
Peri-1 ((−57)–(−22))	5′-CAACGCCACACGCTCCCTTTAGTAGGAGGCGTCCGA-3′
Peri-1 mEts-1	5′-CAACGCCACACGCTCCCTTTAGTAGGAGGCGTCCGA-3′
BPeri-1 ((−88)–(−30))	5′- BIOTIN-GGGACCCGCCCACTGCGCCTGCGCGGCGTGACAACGCCACACGCTCCCGGAAGTAGGAG-3′

For the competition assay, unlabeled double-stranded oligonucleotides were incubated with the nuclear extracts at 4°C for 20 min before adding the labeled probe. For the immunodepletion EMSA, the following procedure was carried out as described previously [91], [92], [93], [94], [95]. Briefly, each aliquot of nuclear extract was incubated with 1 µl of the antibodies and rocked at 4°C for 1 h in 20 µl of EMSA binding buffer. Ten microlitres of protein A/G–Agarose beads pre-washed by EMSA binding buffer were then added and rocked at 4°C for 3 h. The resulting supernatant was subjected to EMSA analysis.

### DNA Affinity Precipitation Assays (DAPAs)

CHO cells were transfected with or without Nc siRNAs. Twelve hours after transfection, the cell lysates were prepared using the Cell Lysis Buffer (Beyotime) with PMSF and sonication. The cell debris was removed by centrifugation (2×5 min, 10,000×*g*, 4°C). The cell extracts, containing 700 µg of protein, were incubated for 16 h with 1 µg of the biotinylated double-stranded oligonucleotides that corresponded to the different regions of the *meri-1* promoter, including BPeri-1 ((−88)–(−30)), BPeri-1 ((−99)–(−64)), and BPeri-1((−57)–(−22)) (see Table 1 for the sequences). The biotinylated DNA-protein complexes were isolated using the µMACS™ Streptavidin Kit (Miltenyi Biotec) according to the manufacturer's instructions. The columns were washed five times and eluted with denaturing buffer. The elutes were then immunoblotted by Sp1, Ets-1 and STAT3 antibodies, respectively.

### Chromatin immunoprecipitation (ChIP) assays

CHO cells were transfected with or without Nc siRNAs. Twelve hours after transfection, ChIP assays were performed using EZ ChIP Kit (Upstate, Biotechnology, Lake Placid, NY) and antibodies against Sp1, Ets-1, STAT3 or control IgG. The input control DNA or immunoprecipitated DNA was then subjected to PCR amplification using primers specific to *meri-1* promoter (Peri-1_ChIP_F: 5′-CACACGCTCCCGGAAGTAG-3′ and Peri-1_ChIP_R: 5′-TCGCTCTCTCCCTTGAAAAA-3′). The annealing sites of these primers are shown in Figure 3F. The PCR products were separated by 2% agarose-gel electrophoresis and visualized with ethidium bromide staining. Each experiment was repeated at least three times.

### Co-immunoprecipitation (Co-IP)

CHO cells were pre-transfected with the FLAG-Sp1 expression plasmid and gene specific siRNAs for twelve hours prior to transfection with Nc siRNA_2. Twelve hours after Nc siRNA_2 transfection, cell lysates were prepared using the Cell Lysis Buffer containing PMSF and TSA (Cell Signaling Technology, Beverly, MA) followed by sonication. The cell extracts, containing 1 mg of protein, were incubated with M2-anti-FLAG antibody-agarose beads (Sigma) for 3 h. Protein-antibody-agarose complexes were precipitated, washed, and examined by Western blotting using indicated antibodies.

### Western blots

Antibodies to the Sp1 and Ets-1 nuclear proteins were purchased from Santa Cruz Biotechnology (Santa Cruz, CA). STAT3 antibody was purchased from Cell Signaling Technology (Beverly, MA). HRP-conjugated FLAG antibody was purchased from Shanghai Westang Bio-tech Co. Ltd. The transfected cell lysate was prepared using lysis buffer containing 50 mM Tris-HCl, pH 6.8, 2% SDS, 10% glycerol, 100 mM dithiothreitol and 0.1% bromophenol blue. The proteins were resolved on 10% SDS-PAGE gels and Western blotted using standard protocols. The primary antibodies were diluted at 1∶500 or 1∶2000, and the AP- or HRP-conjugated, goat anti-mouse or goat anti-rabbit secondary antibody was diluted at 1∶2000 before using.

### Statistical analyses

Results were expressed as mean ± SD. Statistical significance was evaluated for data from three independent experiments using Student's *t*-test. A *p* value of <0.05 was considered to be statistically significant.
